# CMR findings in asymptomatic male HIV patients compared to healthy male controls

**DOI:** 10.1186/1532-429X-14-S1-P175

**Published:** 2012-02-01

**Authors:** Roisin B Morgan, Aisling M Loy, Siobhan O'Dea, Eliz Takacs, Fiona Mulcahy, Caroline Daly

**Affiliations:** 1 Cardiology, St. James's Hospital, Dublin, Ireland; 2 GUIDE, St. James's Hospital, Dublin, Ireland

## Summary

### Aims

To study an asymptomatic group of HIV positive men on antiretroviral drugs and compare them to age and sex matched controls in order to detect underlying cardiovascular disease.

## Background

The increased survival associated with the advent of antiretroviral therapy (ART) for HIV in the mid-1990s has been accompanied by an increase in cardiovascular comorbidity and mortality. Atherothrombosis, myocardial disease, hypertension, diabetes, and pulmonary hypertension have been identified as entities associated with treated disease and the risk of major cardiovascular events is higher than in the uninfected population. HIV patients on ART are at higher risk of developing left ventricular (LV) systolic dysfunction, and more recent studies have additionally demonstrated significantly higher levels of diastolic dysfunction and LV hypertrophy to controls.

## Methods

Methods: Prospective cohort study of asymptomatic HIV positive men on antiretroviral drugs. Baseline demographics, routine biochemistry, fasting lipids and glucose were recorded. A resting 12 lead ECG was taken on all participants. Patients were scanned on a 3T Philips machine with standard protocols as per SCMR guidelines and received weight based intravenous gadolinium. Controls were age and sex matched. Post analysis software was used for LV function, mass and wall thickness. Diastolic function was assessed using phase contrast analysis of mitral inflow to calculate e:a ratio.

## Results

30 HIV positive men were compared with 13 male healthy controls. Mean age for cases vs controls was 50.3 years (+/-9years) vs 41.7 (+/- 6.5 years) respectively. Established cardiovascular risk was prevalent in the HIV population at higher rates than controls: cigarette smoking 35%, hypertension on treatment 29.4%, hyperlipidaemia 44.1% with 29.4% on statin therapy vs 0% of controls who smoked, had hypertension or were on statin treatment. HIV patients had a higher rate of diastolic dysfunction, had greater prevalence of increased wall thickness and had more abnormalities detected, both cardiac and non-cardiac(see table 1).

**Table 1:** MR Findings

	Cases	Controls	P value
LVEF(%+/-SD)	66.2 (+/-6 )	63.9 (+/-7 )	0.31
LMVI(g/m2+/-SD)	59.84(+/- 12.99)	53.24 (+/-14)	0.136
Diastolic dysfunction (no, %)	12(35%)	0(0%)	0.0001
Anteroseptal Wall Thickness(mm+/-SD)	11.22(+/-2)	8.93(+/-2.34)	0.007
Posterolateral Wall Thickness(mm+/-SD)	8.18(+/-1.47)	8.56(+/-1.46)	0.45
Presence of Late Gad enhancement in infarct pattern. No.(%)	2(6%)	0	0.16
Extracardiac Findings	Pericardial cyst Thymoma	0	

## Conclusions

In this asymptomatic population of HIV positive men we found an increased incidence of silent myocardial infarction and increased wall thickness. There was also an increase in diastolic dysfunction compared to age matched controls. These data will be expanded to further investigate whether these findings are due to the effects of long term antiretroviral therapy or as a result of increased prevalence of established cardiovascular risks in this patient cohort.

## Funding

Cardiology and GUIDE Research funding from multiple sources.

